# DLKN-MLC: A Disease Prediction Model via Multi-Label Learning

**DOI:** 10.3390/ijerph19159771

**Published:** 2022-08-08

**Authors:** Bocheng Li, Yunqiu Zhang, Xusheng Wu

**Affiliations:** 1 Department of Medical Informatics, School of Public Health, Jilin University, Changchun 130021, China; 2 Shenzhen Health Development Research and Data Management Center, Shenzhen 518028, China

**Keywords:** disease prediction, multi label learning, disease prevention, deep learning

## Abstract

With the increasingly available electronic health records (EHR), disease prediction has recently gained immense research attention, where an accurate classifier needs to be trained to map the input prediction signals (e.g., symptoms, auxiliary examination results, etc.) to the estimated diseases for each patient. However, most of the current disease prediction models focus on the prediction of a single disease; in the medical field, a patient often suffers from multiple diseases (especially multiple chronic diseases) at the same time. Therefore, multi-disease prediction is of greater significance for patients’ early intervention and treatment, but there is no doubt that multi-disease prediction has higher requirements for data extraction ability and greater complexity of classification. In this paper, we propose a novel disease prediction model DLKN-MLC. The model extracts the information in EHR through deep learning combined with a disease knowledge network, quantifies the correlation between diseases through NodeRank, and completes multi-disease prediction. in addition, we distinguished the importance of common disease symptoms, occasional disease symptoms and auxiliary examination results in the process of disease diagnosis. In empirical and comparative experiments on real EHR datasets, the Hamming loss, one-error rate, ranking loss, average precision, and micro-F1 values of the DLKN-MLC model were 0.2624, 0.2136, 0.2190, 88.21%, and 87.86%, respectively, which were better compared with those from previous methods. Extensive experiments on a real-world EHR dataset have demonstrated the state-of-the-art performance of our proposed model.

## 1. Introduction

With the aging of the population and the increasing awareness of public health care, people’s demands for medical and health services are becoming more and more frequent. With the increase in workload, the amount of information that doctors receive and need to call as the leader of medical services also increases exponentially, so it is very easy to cause medical deviations, such as missed diagnosis and misdiagnosis. Predicting the disease of a patient through an automatic diagnosis model can not only help the hospital to carry out the initial triage and guidance of patients, but also reduce errors in the process of clinical diagnosis, improve medical quality and work efficiency, and reduce medical costs [1]. Meanwhile, In the vast majority of developing countries, due to the imbalance of urban development and the distribution of medical resources, there are great differences in the diagnosis level between doctors. Computer-aided or automatic diagnosis can effectively help patients with the disease early warning and chronic disease screening and reduce the difference in the diagnosis level of doctors [2,3].

Clinical electronic health records (EHR) are a document used by medical institutions to record patients’ condition, clinical treatment, guiding intervention process and final diagnosis and treatment results by means of informatization [4]. In recent years, with the vigorous development of computer related technology, the use of data mining related technology for electronic medical record analysis has become a new direction. An important application of data mining in healthcare is disease prediction where the task is commonly formulated as learning a classifier that infers the prediction results from EHR [5].

According to the number of diseases covered by disease prediction, it can be divided into single disease prediction and multi-disease prediction, corresponding to single label classification (SLC) and multi-label classification (MLC) in machine learning. SLC refers to the data to be classified as having only one category. According to the number of categories, it can be divided into single label two categories and single label multi categories. MLC refers to that each data to be classified belongs to multiple different category labels [6]. In the medical field, a patient often suffers from multiple diseases (especially multiple chronic diseases) at the same time. Therefore, multi-disease prediction is of greater significance for patients’ early intervention and treatment, but there is no doubt that multi-disease prediction has higher requirements for data extraction ability and greater complexity of classification.

Existing multi-label learning algorithms can be divided into problem transformation (PT) and algorithm adaptation (AA) strategies [7]. The PT strategy entails transforming MLC into a series of SLC problems, which can be solved using the existing single-label learning algorithm. PT strategy can be categorized into two schemes: binary relevance (BR) and label powerset (LP). The core of the BR scheme is to transform an MLC problem into multiple binary classification problems in which each binary classifier corresponds to a label to be classified [8,9]. As a conventional multi-label learning strategy, BR is relatively simple and easy to understand, but it completely ignores the correlation between labels, which makes it difficult to achieve the optimal performance of the model. To solve this problem, some scholars have proposed a classifier chain method, which connected constructed classifiers in series and considered the interaction between all tags [10,11]. However, as the number of tags to be classified increases, the number of classifiers constructed by such methods also increases. The LP scheme classifies any number of different label combinations as a new label to treat a problem as a single-label problem [12]. During the classification, this scheme cannot consider the combination of tags that do not appear in the training set [13]. In addition, because the new tags formed by the combination method are associated with only a limited number of instances, the data are very sparse or there is a serious imbalance phenomenon. Therefore, the LP scheme often has a poor application effect when the dataset is large or there are many tags.

The AA strategy entails optimizing and improving the existing single-label learning algorithm to form an improved algorithm or a new algorithm, which can be divided into probability model-based methods (e.g., the MFOM model based on a Bayesian algorithm [14]), support vector machine (SVM)-based methods (e.g., Rank-SVM [15]), decision tree (DT)-based methods (e.g., ML-DT [16]), K-nearest neighbor (KNN)-based methods (e.g., ML-KNN [10]), and ensemble learning-based methods (e.g., BoosTexer [17]). With the further development of computer technology, some deep learning (DL) models have been applied to MLC to achieve certain results. For example, Nam J et al. [18] regarded the MLC problem as the prediction of the target label sequence of the given original text and used a recurrent neural network (RNN) to generate label sequences in turn to obtain the correlation between labels; Yang P et al. [19] improved the sequence generation model (SGM) through the disorder of set decoder to reduce the impact of error tags; Gong J et al. [20] proposed a classification model based on a transformer, which captures text features through multilayer transformer structure and solved the MLC problem using the hierarchical relationship of labels. Some scholars employed a convolutional neural network (CNN) for text feature extraction and exploited cyclic neural networks in sequence data to generate label sequences [21,22]. Nowadays, deep learning has gradually become the mainstream method of text classification because of its strong text extraction ability [23]. However, these algorithms still lack the ability to obtain the semantics of texts in a specialized domain, and it is difficult to capture the high-order correlation between tags only through the tags themselves [6], which limits the performance of classifiers.

In summary, the existing research still has the following limitations: (1) the semantic extraction ability of professional text needs to be further strengthened; (2) in the process of classification, the correlation between tags is not fully considered. Based on this, we propose a novel disease prediction model DLKN-MLC. The contributions of this paper include the following: (1) The model extracts the information in EHR through deep learning combined with a disease knowledge network, quantifies the correlation between diseases through NodeRank, and completes multi-disease prediction; (2) we distinguished the importance of common disease symptoms, occasional disease symptoms and auxiliary examination results in the process of disease diagnosis.

The rest of the paper is organized as follows. Section 2 describes the research datasets and the DLKN-MLC model. Experiments results and evaluation are presented in Section 3. Section 4 discusses the results of the model data experiment and comparative experiment. Section 5 concludes the paper and recommends future works.

## 2. Materials and Methods

### 2.1. Datasets

Our experimental dataset is a real EHR of desensitization in the Department of Gastroenterology provided by a first-class hospital at grade 3 in Shenzhen China, which includes two parts: basic clinical information and clinical diagnosis information. The basic clinical information includes the physical examination, auxiliary examination results, treatment process, outcome, and other information, whereas the clinical diagnosis information is the result of ICD-10 coding by professional coding personnel, including the main diagnosis and coding as well as several other diagnoses and coding. There are 5040 I in total, including 76 different diseases. The average number of Chinese characters pIEHR is 487.75, and the average number of diseases per patient is 3.62. Among them, chronic gastritis (K29.500) occurred the most, with a total of 2958 times, and esophageal hiatal hernia (K44.901) occurred the least, with a total of 76 times.

The sequence annotation software, annotation wizard, was selected as the annotation tool. BIO annotation was a form of sequence annotation: each element was labeled “B,” “I,” or “O,” where “B” represents the beginning of the fragment where the element was located, “I” represents the middle position of the fragment where the element was located, and “O” represents information that does not belong to any type. In addition, according to the relevant clinical guidelines, the Baidu health medical dictionary (https://jiankang.baidu.com/widescreen/home/, accessed on 15 April 2021), and 39 Health Net (http://www.39.net/, accessed on 15 April 2021), we constructed a binary-weighted KN for gastroenterology, which included 182 diseases, 1146 clinical manifestations, and 513 auxiliary examination results.

To exploit the samples in the experimental dataset and consider the reliability of the result evaluation, the experimental dataset was divided into five parts on random average, and the experiment was performed via five cross-validations, i.e., one part was selected as the test dataset, four other parts were selected as the training datasets, and five repeated experiments were performed; the dropout mechanism was introduced. Finally, the test set results of five experiments are used as the data input for multi-label classification. The performance of DLKN-MLC is evaluated with the main and other diagnoses in the EHR as the gold standard.

The experimental environment was set as shown in Table 1. When using NodeRank to complete MLC, D was set to the default parameter of 0.85, and the NR value threshold was bounded by the NR value of “standard disease” in each subnetwork to output all diseases whose NR value was greater than or equal to the NR value of “standard disease”.

### 2.2. DLKN-MLC Model

#### 2.2.1. Overview of DLKN-MLC Model

**Figure 1:**
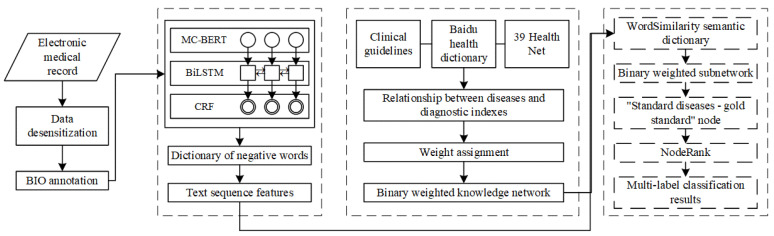
Framework of the DLKN-MLC model.

The framework of the DLKN-MLC model is shown in Figure 1; it includes three main parts. ① DL-based feature extraction: by constructing a feature extraction framework of EHR based on a pretrained word vector, MC-BERT embedded in BiLSTM-CRF (CRF: conditional random field; BiLSTM: bidirectional long short-term memory; MC-BERT: meta controller bidirectional encoder representations from transformers), the semantic acquisition ability is enhanced, and the negative semantic expression is extracted by a negative word dictionary. ② Construction of binary-weighted disease KN: for the ICD-10 disease classification system, the binary-weighted KN between disease and diagnostic indicators is constructed to reflect the correlation between diseases, and different weights are set for incidental symptoms, common symptoms, and auxiliary examinations in diagnostic indicators. ③ MLC based on NodeRank: based on the disease KN, the text sequence features of each patient’s EHR are obtained through DL, and a binary-weighted subnetwork for each patient is constructed. Using NodeRank, the association between each disease label is fully considered, and the disease prediction is completed. The following focuses on these three aspects.

#### 2.2.2. DL-Based Text Sequence Feature Extraction

**Figure 2:**
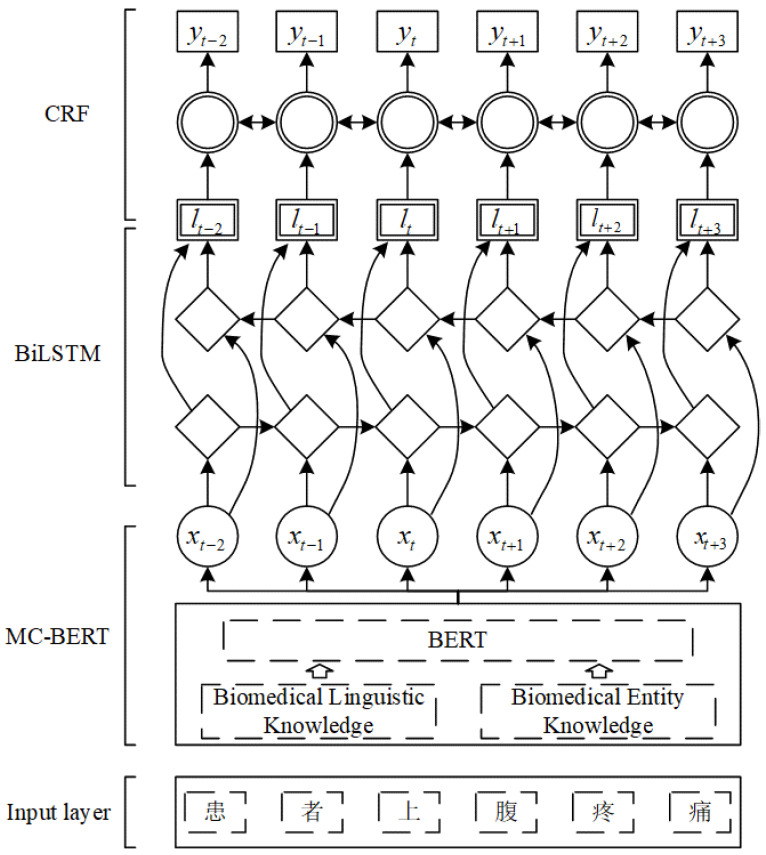
Sequence feature extraction framework based on MCBERT-BiLSTM-CRF. Note: “患者上腹疼痛” means “the patient has epigastric pain”.

The word vector model based on random initialization mainly focuses on the feature extraction of words or between words but ignores the context or semantic information of context. Thus, the extracted vector is separated from the context information, so the effect is general. Therefore, we exploited MC-BERT in semantic representation ability to obtain high-quality word vectors to complete the text sequence feature extraction. Our MCBERT-BiLSTM-CRF sequence feature extraction framework is shown in Figure 2, which has three main modules. First, the MC-BERT pretraining language model is employed to obtain the word vector of the annotated corpus. Compared with the static word vector obtained by conventional pretraining language models, the MC-BERT pretraining language model is trained using a large number of biomedical text corpora; it can exploit context information in a text to generate a word vector to handle polysemy situations efficiently. Then, the word vector is input into the BiLSTM module to further obtain the context information and semantic dependency of the corpus. Finally, the CRF module is used to decode the output of the BiLSTM module to obtain the global optimal tag sequence.

##### MC-BERT

**Figure 3:**
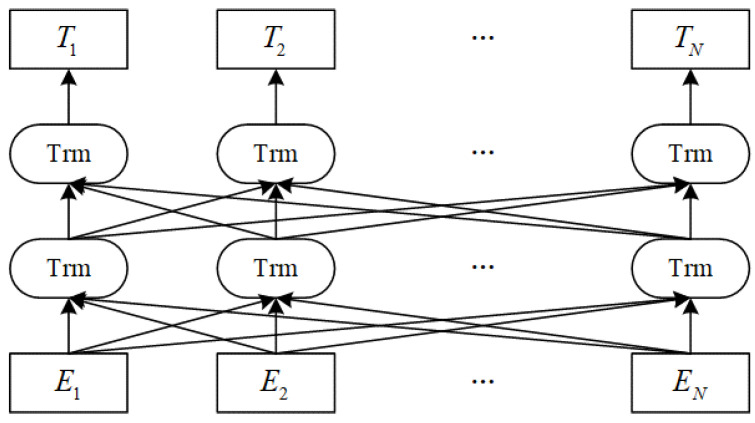
Structure of BERT.

Owing to its excellent semantic representation ability, the BERT pretraining model has achieved tremendous success in related tasks of natural language processing. It can obtain contextualized vectors to improve the extraction performance of text sequence features. The specific structure of the BERT model is shown in Figure 3.

In Figure 3, Trm denotes a self-attention mechanism (transformer) encoding converter$;\;E_{1}$,$\;E_{2}$, …, $E_{N}$ is the input to the model; $T_{1}$, $\;T_{2}$, …, $\;T_{N}$ is the output of the model [24]. The model adopts a multi-layer transformer encoder structure, which can capture the two-way context simultaneously, efficiently characterize the semantic information in the context, obtain more semantic relations, and further enhance the semantic representation ability of vectors.

MC-BERT is a pretraining word vector model proposed by Zhang N et al. [25] for the natural language processing problem in the Chinese biomedical field. Based on the BERT base model, MC-BERT changes the random mask to the medical entity mask and uses the Alibaba cognitive concept map based on biomedicine to mask an entire process to solve the complex structure of Chinese: the problem of multiple combinations of phrases. To enhance the domain applicability of the model, the Chinese medical Q & A, medical encyclopedia, EHR, and other related corpora were used for pretraining.

##### BiLSTM

Long short-term memory (LSTM) is a variant of gradient disappearance or gradient explosion generated by a recurrent neural network (RNN) [26]. LSTM introduces the concept of gating to capture the sequence information of a text and realizes long-term memory. For long texts, such as EHR, which have pre- and post-dependence, its application effect is better than the gated recurrent unit (GRU) model, which is also a variant of RNN [27].

LSTM mainly includes a forget gate, input gate, output gate, and memory cell. The input and forget gates work together to filter useless information and transmit useful information to the next moment. An LSTM network can be formally expressed as Formulas (1)–(6).

(1) $$i_{t}=\sigma \left(W_{xi}x_{t}+W_{hi}h_{t-1}+W_{ci}c_{t-1}+b_{i}\right)$$

(2) $$z_{t}=tanh\left(W_{xc}x_{t}+W_{hc}h_{t-1}+b_{c}\right)$$

(3) $$f_{t}=\sigma \left(W_{xf}x_{t}+W_{hf}h_{t-1}+W_{cf}c_{t-1}+b_{f}\right)$$

(4) $$c_{t}=f_{t}c_{t-1}+i_{t}z_{t}$$

(5) $$o_{t}=tanh\left(W_{xo}x_{t}+W_{ho}h_{t-1}+W_{co}c_{t}+b_{o}\right)$$

(6) $$h_{t}=o_{t}tanh\left(c_{t}\right)$$

where$\;i_{t}$, $f_{t}$, and $O_{t}$ are the output results of input, forget, and output gates, respectively, at time t; $h_{t}$ is the output of the entire LSTM unit at time t; $z_{t}$ means information to be added; σ is the activation function; w is the weight matrix; *b* is the bias vector.

To obtain more information about a text, we exploited the research [2,28] and introduced the bidirectional structure based on conventional LSTM. The bidirectional long, short term memory (BiLSTM) processes each word sequence through forward and reverse LSTMs and completes the output merging simultaneously through the connection layer of the output results. The output is shown in Formula (7):

(7) $$h_{t}=\left[\vec{h_{t}},\overset{\leftarrow }{h_{t}}\right]$$

##### CRF

In sequence feature extraction, BiLSTM has the advantage of processing long-distance text information but fails to consider the dependency between adjacent entities. Therefore, the CRF algorithm [29] is introduced to obtain the global optimal sequence through the relationship between adjacent tags, which compensates for the deficiency of BiLSTM.

For any text sequence, $X=\left(x_{1},x_{2}\ldots ,x_{n-1},x_{n}\right)$, and prediction sequence $Y=\left(y_{1},y_{2}\ldots ,y_{n-1},y_{n}\right)$, let p be the output score matrix of BiLSTM, P∈n∗k, where *n* denotes the number of words in the sequence, *K* denotes the number of tags in the sequence, and the function formula is shown in Formula (8).

(8) $$s\left(X,Y\right)=\overset{n}{\underset{i=0}{\sum }}A_{yi,yi+1}+\overset{n}{\underset{i=1}{\sum }}P_{i,yi}$$

where *A* represents the transfer fraction matrix, the size is K + 2, $A_{ij}$ represents the score of label I transferred to label *j*, $P_{ij}$ is the score of the *j*-th tag of the *i*-th word. The probability formula of prediction sequence y is shown in Formula (9).

(9) $$P\left(Y|X\right)=\frac{e^{s\left(X,Y\right)}}{\sum_{\tilde{Y}\in Y_{X}}s\left(X,\tilde{Y}\right)}$$

Taking the logarithm of both sides to obtain the likelihood function of the prediction sequence, we have Formula (10)

(10) $$ln\left(p\left(Y|X\right)\right)=S\left(X,Y\right)-ln\left(\underset{\tilde{Y}\in Y_{X}}{\sum }s\left(X,\tilde{Y}\right)\right)$$

where $\tilde{Y}$ represents the real annotation sequence, and $Y_{X}$ represents all possible annotation sequences. The formula of the output sequence to obtain the maximum score after decoding is as follows:

(11) $$Y^{*}=argmax\;s\left(X,\tilde{Y}\right)\;\;\tilde{Y}\in Y_{X}$$

Therefore, we combined CRF with BiLSTM to obtain the global optimal marker sequence.

Further, in addition to the direct expression of related diseases and examinations, there are negative expressions of negative words on semantics, such as no palpitation and no abdominal mass. To avoid the interference of this part of the information in the final classification, we constructed a negative word dictionary containing 46 negative words by referring to the modern Chinese dictionary and previous research [30]. Combining it with the global optimal marker sequence obtained by DL, the first, second, and third parts of the marker sequence were analyzed. The negative words in the last two characters and those in the marker sequence were regarded as negative words negating the semantics in their jurisdiction, forming a sequence marker containing a negative semantic relationship.

#### 2.2.3. Construction of Binary-Weighted Disease KN

The correlation between diseases in the medical field is more complex than in other fields. For example, for news, entertainment news is less likely to be related to politics; for sentiment analysis, the emotional expression of “happiness” and “joy” often appears in the same comment [31]. However, the comorbidity of patients has a certain relationship with the population and region of disease [32]. It is not a simple linear correlation, and it is difficult to be reflected by the label data itself. Therefore, to efficiently obtain the high-order correlation between diseases, we constructed a binary-weighted KN between diseases and diagnostic indicators and reflected the correlation between diseases through the correlation between diagnostic indicators and diseases. This study is based on a confirmed conclusion in the medical field: “if two diseases have the same or similar clinical manifestations, they may have the same pathogenic mechanism and genetic basis.” [33]. Based on the related theory of complex networks, from the perspective of network topology analysis, the binary-weighted KN G (D,D’) for patients’ clinical manifestations; auxiliary examinations more intuitively describe the relationship between diseases and diagnostic indicators. We use nodes of different shapes to represent diseases, auxiliary examination results, or clinical manifestations of diseases. The connection between nodes indicates that the clinical manifestation or auxiliary examination results can support the disease diagnosis. The number on lines indicates the strength of the contribution to the diagnosis. We set the corresponding weights for different diagnosis indicators. See Section 3.2 for the weight setting process and results.

#### 2.2.4. MLC Based on NodeRank

Based on the constructed binary-weighted KN, the clinical manifestations and auxiliary examination results are extracted from the text sequence features of a patient’s EHR, and the clinical manifestations and negative examination results containing negative expressions are removed for matching in the binary-weighted KN, A binary-weighted subnetwork including all clinical manifestations, auxiliary examination results, and possible associated diseases was formed. In addition, because the number of tags assigned to each EHR is different in multi-label classification, to further determine the output threshold of multi tags we added a “standard disease–gold standard” relationship in each subnetwork to distinguish the comprehensive contribution of diagnostic indicators to diseases, which comprises a standard disease node and a gold standard node. The gold standard node represents the only gold standard used to diagnose standard diseases. It is connected with only standard diseases, and not related to other diseases. The weight setting is the same as that of auxiliary examination results. This relationship means that the contribution of the diagnostic criteria connected with a disease reaches the level of a “gold standard”.

Owing to the poor standardization of writing EHR of some doctors, the use of words is not unified, and there are many similarities between some clinical manifestations and auxiliary examination words. There are some differences between the expression and the diagnosis and treatment guidelines, such as “腹痛 (abdominal pain)” and “腹部疼痛 (abdominal pain)”. Therefore, we introduce WordSimilarity semantic dictionary (https://wordsimilarity.com/, accessed on 18 May 2021) to assist in matching words that cannot be filled directly. The matching pseudocode is as follows:

Matching rules for an EHR

The total number of diagnostic indexes included in the case was extracted, m, I = 1

WHILE i = m

{

Extract the i-th diagnostic index in the EHR

If the i-th diagnostic index can match the diagnostic index in KN

from the KN, the diagnosis index and the weight of all diseases and side links are extracted

Else uses WordSimilarity semantic dictionary to match the diagnosis indexes in KN according to the principle of high similarity coefficient first

End if

i = i + 1

}

NodeRank is an improved sorting algorithm based on PageRank with edge weight [34]. The algorithm is based on the idea that “the more links to web pages, the higher the importance of the web pages”. Similarly, if multiple clinical manifestations or auxiliary examination results of a patient are related to a disease simultaneously, the more likely the patient is to develop the disease. The specific formula of NodeRank is as follows [35]:

(12) $$NR\left(D\right)=\left(1-d\right)+d\overset{i}{\underset{i=1}{\sum }}\frac{w\left(f_{i}\cdot D^{\prime }\right)}{\sum_{j=1}^{m}w\left(f_{i}\cdot f_{j}\right)}NR\left(f_{i}\right)$$

where *D* is the Gini coefficient of a binary-weighted subnetwork, NR(D) is the importance of the disease in the patient’s binary network, $w\left(f_{i}\cdot D^{\prime }\right)$ denotes the weight that points to the edge of the disease, and $\overset{m}{\underset{j=1}{\sum }}w\left(f_{i}\cdot f_{j}\right)$ indicates the weight of all-out edges of the diagnostic index. In this study, MLC is considered to be a ranking problem and uses NR(D) to complete the classification [36]. The higher the value of NR(D), the higher the probability of patients having the disease.

## 3. Results

### 3.1. Evaluation Metrics

To evaluate the performance of DLKN-MLC, we selected five widely used MLC evaluation metrics, Hamming loss (HL), one-error rate (OE), ranking loss (RL), and average precision (AP), and micro-F1 [37,38,39]. We also compared the proposed model with comparison models.

Let $D=\left\{x_{i},y_{i}|1\leq i\leq N\right\}$ be a multilabel test set, $x_{i}$ represents the EHR text in the test set, $y_{i}\;$is the real label corresponding to $x_{i}$, *N* is the number of samples in the test set, $Y_{i}$ represents the set of label spaces for the dataset, *Q* is the total number of labels in the label space set, h(·) is the multi-label classifier, and $h\left(x_{i}\right)\;$is the prediction result of the sample $x_{i}$ in the test set.

Hamming loss (HL) is the proportion of inconsistency between the predicted and real tags. The calculation is shown in Formula (13), where $h\left(x_{i}\right)∆y_{i}$ is the number of real tag sets different from predicted tag sets.

(13) $$HL=\frac{1}{N}\overset{N}{\underset{i=1}{\sum }}\frac{1}{Q}\left|h\left(x_{i}\right)∆y_{i}\right|$$

One-error (OE) indicates the probability that the tag with the highest prediction probability is not in the real tag set. The calculation is shown in Formula (14), where $argmax\;h\left(x_{i}\right)$ indicates the label with the highest prediction probability.

(14) $$OE=\frac{1}{N}\overset{N}{\underset{i=1}{\sum }}\left[\left[argmax\;h\left(x_{i}\right)\right]\notin Y_{i}\right]$$

Ranking loss (*RL*) is the average number of times that wrong tags appear before correcting tags in the ranking sequence of the prediction tag set, given by Formula (15).

(15) $$RL=\frac{1}{N}\times \overset{N}{\underset{i=1}{\sum }}\frac{\left|\left\{\left(h\left(x_{i}\right),y_{i}\right)\left|f\left(x_{i},h\left(x_{i}\right)\right)\right|\leq f\left(x_{i},y_{i}\right),\left(h\left(x_{i}\right),y_{i}\right)\in y_{i}\times \bar{y_{i}}\right\}\right|}{\left|y_{i}\right|\left|\bar{y_{i}}\right|}$$

where $\bar{y_{i}}$ is the complement of the real label set $y_{i}$ in the label space, and f (·) is the prediction value generated by the multi-label classifier.

Average precision (*AP*) is the average number of correct sorting in the prediction tag. The calculation is shown in Formula (16), where *rank* (·) is the sorting function.

(16) $$AP=\frac{1}{N}\overset{N}{\underset{i=1}{\sum }}\frac{1}{Q}\underset{h\left(x_{i}\right)\in Y_{i}}{\sum }\frac{\left|\left\{y_{i}|rank\left(f\left(x_{i}\right),y_{i}\right)\leq rank\left(f\left(x_{i}\right),h\left(x_{i}\right)\right),y_{i}\in Y_{i}\right\}\right|}{rank\left(x_{i},h\left(x_{i}\right)\right)}$$

Micro-F1 represents the harmonic average value of micro precision and micro recall considering the overall situation of all labels. It can better reflect the overall performance of the sample under the real distribution. The calculation is shown in Formulas (17)–(19). Let a confusion matrix be generated for a certain type of label as Table 2.

(17) $$Micro-precision=\frac{\sum_{j=1}^{Q}TP_{j}}{\sum_{j=1}^{Q}\left(TP_{j}+FP_{j}\right)}$$

(18) $$Micro-recall=\frac{\sum_{j=1}^{Q}TP_{j}}{\sum_{j=1}^{Q}\left(TP_{j}+FN_{j}\right)}$$

(19) $$Micro-F1=\frac{\sum_{j=1}^{Q}2TP_{j}}{\sum_{j=1}^{Q}\left(2TP_{j}+FP_{j}+FN_{j}\right)}$$

### 3.2. Comparison Results of Different Weighting Values

Through consulting relevant clinical experts and facing the weight setting in the binary-weighted KN, five groups of different weight combinations were set for the incidental clinical manifestations, common clinical manifestations, and auxiliary examination results of diseases (Table 3). Data experiments were performed for these five groups of weight combinations; the results are shown in Table 4.

Therefore, based on the above five groups of experimental results, the odd weight setting scheme was selected, with occasional clinical symptoms set to 1, common clinical symptoms set to 3, highlighting the strong evidence and depth of auxiliary examination results, and the weight set to 7, to distinguish the contribution of different information to disease diagnosis and further improve the model performance.

### 3.3. Experimental Results

**Figure 4:**
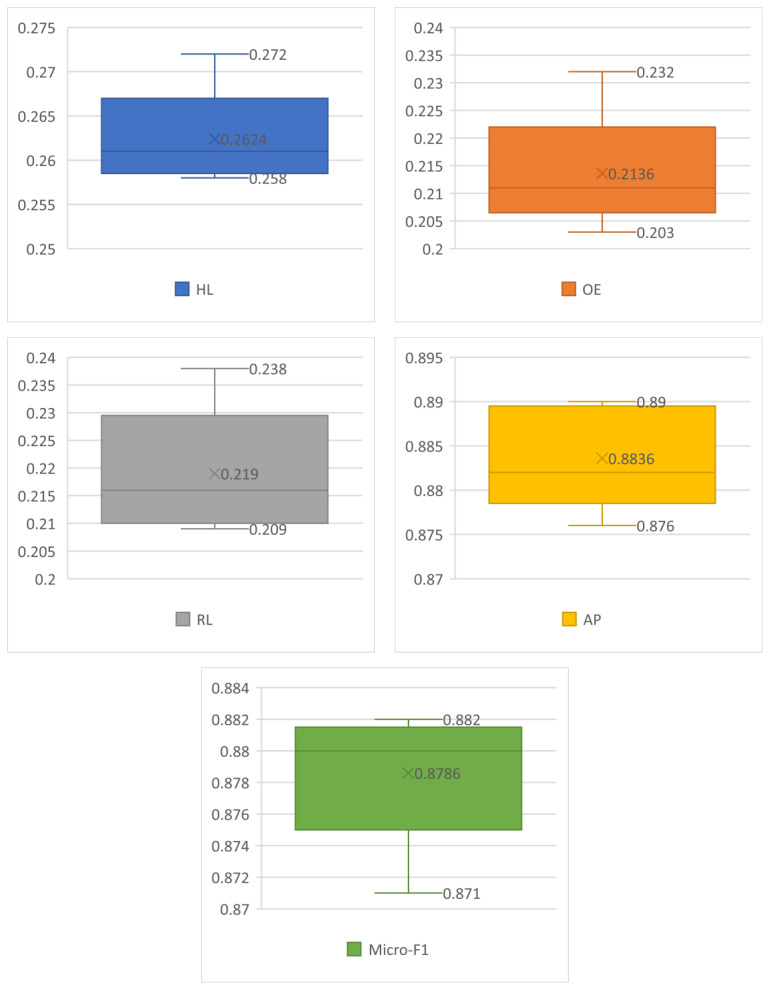
Model experimental results.

The experimental results are shown in Figure 4, in which HL is 0.2624, OE is 0.2136, RL is 0.2190, AP is 0.821, Micro-F1 is 0.8786, and the relevant indicators perform well. At the same time, we can find that the performance of each indicator is also relatively stable through each box plot.

## 4. Discussion

### 4.1. Influence of Different Weights on Model Performance

In Table 3, by comparing groups <113> and <123>, we found that the performance of the model could be improved by distinguishing the incidental clinical manifestations and common clinical manifestations of diseases. This was because reducing the weight of the incidental clinical manifestations of the disease could reduce the impact of the same or similar symptoms among the diseases and further improve the accuracy and ranking loss of the model. By comparing groups <135> and <137>, we found that increasing the weight of auxiliary diagnosis results was helpful to further improve the model performance. The auxiliary diagnosis results are often the in-depth examination of diseases using specific instruments, which have greater reference values for disease diagnosis. In addition, the weight of auxiliary diagnosis results in this study is the same as that in the “standard diseases-gold standard”. Increasing the weight can reduce the output of low-ranking results and improve the accuracy of the model. In the comparison of groups <135>, <137>, and <139>, we found that increasing the weight value of auxiliary diagnosis results would improve the OE and RL of the model, but when the weight was too large, the performance of the model in terms of HL, AP, and micro-F1 value declined, which was because increasing the weight could reduce the output of low-ranking results and improve the accuracy of the model. However, when the weight was too large, the accuracy and completeness of the model were out of balance, and the model eliminated too many diseases with a relatively low ranking.

Therefore, based on the above five groups of experimental results, the odd weight setting scheme is selected, with occasional clinical symptoms set to 1, common clinical symptoms set to 3, highlighting the strong evidence and depth of auxiliary examination results, and the weight set to 7, in order to distinguish the contribution of different information to disease diagnosis and further improve the performance of the model.

### 4.2. Comparison Algorithm Selection

To further verify the DLKN-MLC model, we selected the representative algorithm Text-CNN [40], CNN–RNN [41], and X-BERT [42] as comparison models. The same method of five cross-validations was used for testing in this dataset. Table 5 shows the information of the comparison model.

### 4.3. Analysis of Comparison Model Results

The comparison model results are shown in Table 6. By comparing the relevant indicators, we found that the DLKN-MLC model was better than the comparison model. Its AP was 88.21%, which was 2.93% higher than that of X-BERT.

Outstanding results are closely related to the introduction of binary-weighted KN to comprehensively consider the correlation between diseases. Although X-BERT also uses the dependency relationship between labels, compared with other fields, the medical field is affected by the limitation of sample size and disease complexity. It is difficult to learn the model and reflect the correlation and dependence between diseases. The HL, OE, and RL values of the proposed model were 0.5624, 0.2136, and 0.2190, respectively, which were better than those of X-BERT and CNN–RNN. The superiority in ranking related indicators highlights the efficiency of using NodeRank with edge-connected weight to sort and classify texts and using the “standard disease–gold standard” relationship to control the output nodes. The combined use of the two not only distinguished the contribution of different diagnostic indicators to the diagnosis but also controlled the output well. In the comprehensive index micro-F1 value, 87.86% of the research results were also better than those of comparison models, which proved the comprehensive advantages of the proposed extraction framework from text feature extraction to final sorting output.

At the same time, most of the current studies are end-to-end models [43]; compared with such studies, the DLKN-MLC model has better interpretability [44]. We use the DLKN-MLC model to simulate the process of doctors obtaining patient information and making diagnosis inferences, and we can obtain the information of DLKN-MLC used to infer disease through the matching information of patient disease information extracted from the EHR and binary-weighted network. It is not a black box model. Furthermore, our model distinguishes the importance of common disease symptoms, accidental disease symptoms and auxiliary examination results in the process of disease diagnosis through weights, which enables us to adjust the specificity and sensitivity of the model to a certain extent, so that it can be applied to different scenarios, such as large-scale disease screening and diagnosis [45]. Compared with the abstract hyperparameters in traditional deep learning, this is more conducive to user understanding.

## 5. Conclusions

In conclusion, we proposed a novel disease prediction model based on a Chinese EHR named DLKN-MLC. The model extracts the features of EHR through the DL module, uses the binary-weighted KN to obtain the correlation between diseases, and then uses NodeRank to complete the final sorting classification. The results showed that the model could further improve the performance of disease prediction. We also verified the effectiveness and superiority of DLKN-MLC, which had certain methodological significance.

However, there are still some limitations in this paper: the DLKN-MLC model is discussed from the influence of different weighting values and the quality of model prediction, and the running cost and time efficiency of the model are not discussed. In future research, we will compare and analyze the complexity and running time of relevant models and consider using the public disease knowledge graph for auxiliary classification, so as to further enhance the correlation between diseases.

## Figures and Tables

**Table 1:** Experimental environment setting.

Experimental Environment	Experimental Configuration
GPU	GTX 1050TI
CPU	E5-2678V3
Development environment	Python3.7.3 TensorFlow1.15.2
Epoch	20
Optimizer	Adam
LSTM learning rate	0.001
Dropout	0.5

**Table 2:** Confusion matrix.

Confusion Matrix		Predictive Value	
		Positive	Negative
Actual value	Positive	TP	FN
	Negative	FP	TN

**Table 3:** Weight setting group of binary-weighted KN.

Group	<113>	<123>	<135>	<137>	<139>
occasional clinical manifestations	1	1	1	1	1
common clinical manifestations	1	2	3	3	3
auxiliary diagnostic results	3	3	5	7	9

**Table 4:** Experimental results of weight setting of five groups (MEA ± SD).

	HL↓	OE↓	RL↓	AP↑	Micro-F1↑
<113>	0.3076 ± 0.005634	0.2412 ± 0.008233	0.3153 ± 0.009842	0.8623 ± 0.005285	0.8496 ± 0.003933
<123>	0.2966 ± 0.005754	0.2357 ± 0.009018	0.2962 ± 0.009632	0.8642 ± 0.005021	0.8522 ± 0.003828
<135>	0.2687 ± 0.004982	0.2257 ± 0.008721	0.2276 ± 0.010223	0.8786 ± 0.004692	0.8672 ± 0.003468
<137>	**0.2624 ± 0.005004**	0.2136 ± 0.009728	0.2190 ± 0.010373	**0.8821 ± 0.004782**	**0.8786 ± 0.003587**
<139>	0.2695 ± 0.005229	**0.2085 ± 0.009536**	**0.2162 ± 0.010648**	0.8754 ± 0.004724	0.8654 ± 0.003622

Note: The bold value in the table is the optimal value under the index; “**↑**” means that the larger the index is, the better the classification effect is; “**↓**” means that the smaller the index is, the better the classification effect is.

**Table 5:** Introduction to comparison model.

Comparison Model	Model Description
Text-CNN	On the basis of CNN, many sliding windows of different sizes are added, and the feature extraction is carried out by a convolution kernel.
CNN-RNN	CNN and RNN are combined to extract the local features of the text, and RNN is used to obtain the sequence features and high-order tag correlation of the text.
X-BERT	At the same time, tags and input text are used to generate semantic tag clusters to make better use of the dependency relationship between tags for modeling.

**Table 6:** Model performance comparison results (MEA ± SD).

	HL↓	OE↓	RL↓	AP↑	Micro-F1↑
Text-CNN	0.3672 ± 0.009621	0.3112 ± 0.008635	0.2922 ± 0.013585	0.7838 ± 0.005145	0.7838 ± 0.005785
CNN–RNN	0.2914 ± 0.006888	0.2598 ± 0.009537	0.2454 ± 0.008639	0.8204 ± 0.005848	0.8058 ± 0.007243
X-BERT	0.2788 ± 0.006675	0.2412 ± 0.006431	0.2494 ± 0.009972	0.8528 ± 0.007514	0.8362 ± 0.004946
Our method	**0.2624 ± 0.005004**	**0.2136 ± 0.009728**	**0.2190 ± 0.010373**	**0.8821 ± 0.004782**	**0.8786 ± 0.003587**

Note: The bold value in the table is the optimal value under the index; “↑” means that the larger the index is, the better the classification effect is; “↓” means that the smaller the index is, the better the classification effect is.

## Data Availability

The data presented in this study are available on request from the corresponding author.
